# Pain catastrophising as a risk factor for hospitalisation and readmissions in fast-track hip and knee arthroplasty, an observational multicentre cohort study

**DOI:** 10.1016/j.bjao.2026.100564

**Published:** 2026-06-01

**Authors:** Christoffer C. Jørgensen, Simon Kornvig, Henrik Kehlet, Martin Lindberg-Larsen, Kirill Gromov, Manuel J. Bieder, Mikkel R. Andersen, Søren Overgaard, Torben B. Hansen, Thomas Jakobsen, Claus Varnum

**Affiliations:** 1 The Centre for Fast-track Hip and Knee Replacement, Denmark; 2 Department of Anaesthesia and Intensive Care, Copenhagen University Hospital – North Zealand, Hillerød, Denmark; 3 Department of Clinical Medicine, University of Copenhagen, Copenhagen, Denmark; 4 Department of Orthopaedic Surgery, Lillebaelt Hospital - Vejle, University Hospital of Southern Denmark, Vejle, Denmark; 5 Section for Surgical Pathophysiology, Copenhagen University Hospital- Rigshospitalet, Copenhagen, Denmark; 6 Department of Orthopaedic Surgery and Traumatology, Odense University Hospital, Svendborg, Denmark; 7 Department of Orthopaedic Surgery, Copenhagen University Hospital - Amager and Hvidovre, Hvidovre, Denmark; 8 Department of Orthopaedic Surgery, Naestved-Slagelse-Ringsted Hospitals, Naestved, Denmark; 9 Department of Orthopaedic Surgery, Copenhagen University Hospital - Herlev and Gentofte, Herlev, Denmark; 10 Department of Orthopaedic Surgery and Traumatology, Copenhagen University Hospital – Bispebjerg and Frederiksberg, Copenhagen, Denmark; 11 Department of Orthopaedic Surgery, Gødstrup University Hospital, Gødstrup, Denmark; 12 Department of Clinical Medicine, Aarhus University, Aarhus, Denmark; 13 Department of Orthopaedic Surgery, Aalborg University Hospital, Farsø, Denmark

**Keywords:** ERAS, fast-track surgery, hip arthroplasty, knee arthroplasty, pain catastrophising, perioperative medicine, postoperative pain

## Abstract

**Background:**

A preoperative pain catastrophising scale (PCS) of >20 is associated with increased postoperative pain, development of chronic pain, and impaired function after hip and knee arthroplasty. However, whether PCS >20 is associated with prolonged length of hospital stay (LOS), readmissions, and reduced likelihood of same-day discharge is unknown.The primary aim of this study was to investigate the association between a preoperative PCS of >20 and a LOS >2 days Secondary aims were to investigate the association of PCS >20 with 90-day readmission rate and admission postoperatively.

**Methods:**

This prospective cohort study is from eight Danish arthroplasty departments with similar fast-track protocols. Data on preoperative PCS, patient characteristics, and prescribed medications were collected. Postoperative follow-up was conducted through patient records and self-reported questionnaires. Binary multilevel logistic regression was used to investigate the association between PCS >20 and hospital LOS of >2 days, 90-day readmission rate due to pain or insufficient mobilisation, and immediate admission despite scheduled same-day surgery.

**Results:**

The study period ran from September 2022 to June 2025. Of 15 965 procedures, 6728 (43%) were total hip arthroplasties (THA), 5992 (38%) were total knee arthroplasties (TKA), and 2974 (19%) were unicompartmental knee arthroplasties (UKA). Of these, 5225 (33%) reported a preoperative PCS >20. Median hospital LOS was 1 day, 90-day readmission rate was 7.3%, and 5464 (35%) of patients were scheduled for same-day discharge. For the total cohort, PCS > 20 was associated with LOS >2 days (odds ratio [OR]: 1.54; 95% confidence interval [CI]: 1.29–1.83; *P*<0.001), 90-day all-cause readmissions (OR: 1.18; CI:1.03–1.35; *P*=0.02), hospitalisation due to pain or insufficient mobilisation (OR: 1.38; CI: 1.11–1.72; *P*=0.004), and admission despite planned same-day discharge (OR: 1.25; CI: 1.06–1.40; *P*=0.005). However, procedure-specific analysis did not find associations with LOS after UKA (OR: 0.59; CI: 0.30–1.17; *P*=0.129), or readmission after TKA (OR: 1.02; CI: 0.82–1.26; *P*=0.890).

**Conclusions:**

A preoperative pain catastrophising scale of >20 was associated with increased hospital length of stay and increased 90 day readmission rate despite planned same-day discharge after fast-track total hip arthoplasty, total knee arthoplasty and unicompartmental knee arthroplasty surgery.

**Editor’s key points**

• Postoperative pain remains a challenge after fast-track hip and knee arthroplasty, potentially leading to prolonged hospitalisation and readmission.
• This observational multicentre cohort study from eight major Danish arthroplasty centres with well-established fast-track and same-day surgical protocols investigated the association between a preoperative pain catastrophising scale (PCS) of >20, length of stay (LOS) >2 days, and readmission.
• The authors report that a PCS >20 was associated with increased hospital LOS >2 days and 90-day all-cause readmissions after fast-track hip and knee arthroplasty, with these outcomes specifically due to pain or mobilisation issues.
• Pain catastrophising is a risk factor for hospitalisation and readmissions in fast-track hip and knee arthroplasty. Future interventional studies could consider targeted attention to those with a preoperative PCS >20, as these patients may benefit the most from pain-preventative interventions.

Hip and knee arthroplasty are common major orthopaedic procedures to alleviate pain and improve function and quality of life in patients with advanced osteoarthritis. Currently, the development of enhanced recovery after surgery (ERAS) or fast-track protocols has improved perioperative care, leading to reduced postoperative morbidity, resulting in a significant decrease in length of hospital stay^1^ (LOS) and utilisation of same-day surgical procedures.^2^ However, despite several evidence-based analgesic regimens,^3^^,^^4^ postoperative pain remains a challenge, potentially leading to prolonged hospitalisation^5^ and readmission.^1^

In recent decades, it has been increasingly acknowledged that preoperative psychological characteristics may influence the individual response to injury, with patients called ‘pain catastrophisers’ being more likely to experience exaggerated levels of acute postoperative pain.^6^^,^^7^ One way of identifying such patients preoperatively is by using the pain catastrophising scale (PCS),^8^ which has repeatedly demonstrated an association with both postoperative acute pain, development of persistent pain, and impaired long-term functional outcomes after all types of major joint replacement.^6^^,^^9^, ^10^, ^11^ However, most studies have been performed in patients having surgery prior to the most recent evidence-based ERAS or fast-track protocols and with varying analgesic treatment.^11^ Within fast-track hip and knee arthroplasty, a PCS of >20 has been used to select patients for several randomised clinical trials investigating different adjuvant analgesic interventions,^12^, ^13^, ^14^, ^15^ but primarily focusing on acute postoperative pain at 24 h. However, as median LOS after these procedures is currently less than 24 h, and with an increasing proportion of patients having same-day discharge, the question of whether a PCS of >20 is associated with increased LOS, readmissions, or inability to be discharged on the day of surgery within a fast-track setup is of increasing interest in relation to suitability for same-day surgery and potential additions to postoperative analgesic treatment.^16^ The primary aim of this study was to use a prospective database from eight Danish centres with well-established fast-track and same-day surgical protocols^17^ to investigate the association between a preoperative PCS of >20 and a LOS >2 days. Secondary aims were to investigate the association of PCS >20 with 90-day readmission rate with having a LOS >2 days or 90-day readmission rate specifically due to pain or insufficient mobilisation, and to investige the relationship between PCS >20 with admission despite being planned for same-day discharge. We hypothesised that a PCS of >20 would be significantly associated with the above-mentioned outcomes, also after adjusting for preoperative characteristics and other potential confounders.

## Methods

### Study design

This is a prospective multicentre observational study performed in eight major Danish arthroplasty centres participating in the Center for Fast-track Hip and Knee Replacement Collaboration. As this is a non-interventional study, it was exempt from approval by the Danish National Ethics Committee (j.nr. 2500557). Reporting is done according to the STrengthening the Reporting of OBservational studies in Epidemiology (STROBE) statement. All patients provided written consent for evaluation of electronic healthcare records and separate consent for receiving questionnaires with an opt-out possibility. The centres participating in the collaboration represent all Danish Healthcare Regions and account for approximately 40% of all annual major arthroplasty procedures in Denmark.^17^ All departments adhere to similar fast-track protocols, including opioid-sparing analgesia, preferred spinal anaesthesia, aligned inclusion criteria for same-day pathways, functional discharge criteria, and discharge to own home.^18^ A single dose preoperative high-dose glucocorticoid consisting of either 24 mg dexamethasone or 125 mg of methylprednisolone is standard in all departments, but in one of department this is increased to 1 mg kg^−1^ in patients with a PCS of > 20 scheduled for total knee arthroplasty (TKA). The use of peripheral nerve blocks also varies between departments, with one department using adductor canal blocks (ACB) as standard in all patients undergoing knee arthroplasty, four departments using ACB upon indication, and two departments practically never using ACB. No department uses peripheral nerve blocks after total hip arthroplasty (THA) as standard.^19^

From September 2022 to October 2025, the collaboration established a detailed database on pre- and postoperative data, including preoperative patient characteristics, laboratory results, medication and functional level, inclusion in same-day surgical pathways, postoperative outcomes, and 90-day follow-up on readmissions and mortality. Data were acquired using a combination of patient-reported questionnaires and review of electronic healthcare records, with a completeness rate of >95% of all performed procedures being assured through regular internal audits. Ninety days after surgery, all patients had their healthcare records reviewed by dedicated healthcare staff, and in cases of admission for scheduled same-day surgical procedures, LOS of > 2 days, 90-day readmissions or 90-day mortality, the primary cause of morbidity was recorded and a short clinical summary detailing the course and complications was extracted. All summaries were re-evaluated by the first author (CJ) to ensure accuracy and consistency of classification. Follow-up is 100% within the same healthcare region and with the possibility for acquiring information from the other regions in the rare cases of admission to another healthcare region. All data are stored in the Center for Fast-track Hip and Knee Replacement database (FTHK) using a Research Electronic Data Capture (REDCap)^20^ database approved by the Region of Southern Denmark (J-no- 22/39454). The database, its scientific aims and methodology are registered on ClinicalTrials.gov (NCT05613439).

### Patients

The study period ran from 9 September 2022 to 6 June 2025. As one department did not register PCS in the FTHK until 15 December 2023, we excluded patients undergoing surgery at this specific department prior to this. There are no formal exclusion criteria for registration in the FTHK, and adherence to the fast-track protocol is the standard of care regardless of whether patients have THA, TKA or UKA. There are formal exclusion criteria for the same-day surgical pathways, which have been detailed previously.^17^ Although revisions and simultaneous bilateral procedures are also registered in the FTHK, we included only patients undergoing unilateral primary elective procedures for this specific study. Highly specialised procedures, including cancer surgery or procedures due to congenital disorders, are not registered in the FTHK. Patients who consented to receive questionnaires completed the PCS questionnaire prior to surgery. The PCS has been validated in Danish^21^ and consists of 13 items regarding how an individual copes with painful stimuli using a 5-point Likert scale, summarised in a score of 0–52.^8^ Patients with an accumulated score of >20 to 30 can be considered ‘pain catastrophisers’ in general,^8^ but with threshold of >20 specifically in major joint arthroplasty.^10^

### Outcomes

The primary outcome was the association between a PCS >20 and a LOS >2 days. Secondary outcomes included 90-day readmission rate, a LOS >2 days or readmissions directly related to pain or insufficient mobilisation, and admission despite planned same-day surgery.

### Sensitivity analysis

As a sensitivity analysis, we conducted a procedure-specific analysis of the primary and secondary outcomes. We also report the outcomes for patients with missing data for PCS separately to investigate potential selection bias in patients without reported PCS. Finally, to evaluate the robustness of our results for the primary outcome, we evaluated the influence of unmeasured confounders with odds ratios of 1.10 and 2.00.^22^

### Power analysis and statistics

A statistical analysis plan, including a power analysis and definition of study endpoints, was written in June 2025, prior to data analysis, and agreed upon by all authors.

A pre-study power analysis was conducted in Gpower version 3.1.9.2. using an a priori z-test for logistical regression with a power of 80% and a significance level of 0.05. We assumed that PCS >20 contributed 10% to the likelihood of having LOS >2 days (R^2^ of 0.90). The assumed fraction of patients with PCS >20 and the fraction with LOS >2 days were set to 27% and 4%, respectively, according to tentatively extracted data. Based upon these assumptions, a total sample size of 13 161 patients would be needed.

The inclusion of adjusted confounders was based on current literature regarding risk factors for postoperative pain and increased LOS, using directed acyclic graphs^23^ available from https://www.dagitty.net/. Logistic regression using a hierarchical model with department of surgery as a random effect was conducted while adjusting for type of procedure (THA/TKA/UKA), age, sex, clinical frailty score, planned same-day surgery, cardiac medication, preoperative opioids, pulmonary medication, psychotropic treatment, use of walking aids, preoperative anaemia (<13 g dl^−1^ regardless of sex), diabetes, and cohabitation. Cases with missing data for individual variables (<5% of patients) were excluded from the logistic regression analysis. The code used can be found in the statistical analysis plan (Supplemental Digital Content 1).

Proportions are reported as percentages with 95% confidence intervals (CI). Continuous variables are reported as medians with interquartile ranges (IQR) or means with standard deviations, as appropriate. A *P*<0.05 was considered significant. Analyses were performed using SPSS version 29.0.1.0 (IBM Corporation, Armonk, NY, USA).

## Results

**Fig 1:**
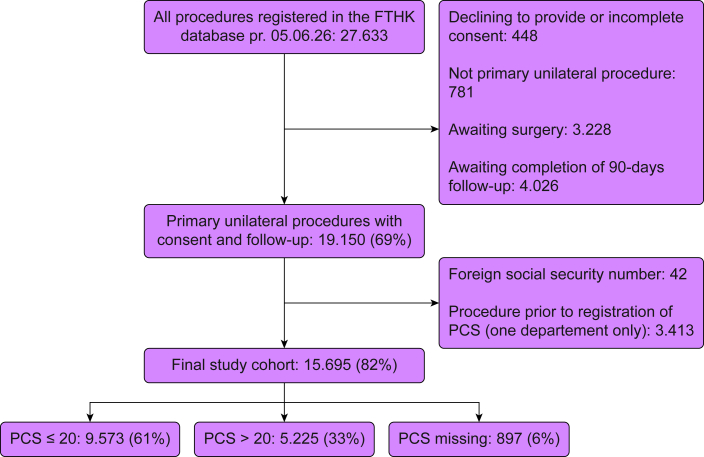
CONSORT diagram of the study population. FCHK: Center for Fast-track Hip and Knee Replacement. PCS: Pain catastrophizing scale.

**Fig 2:**
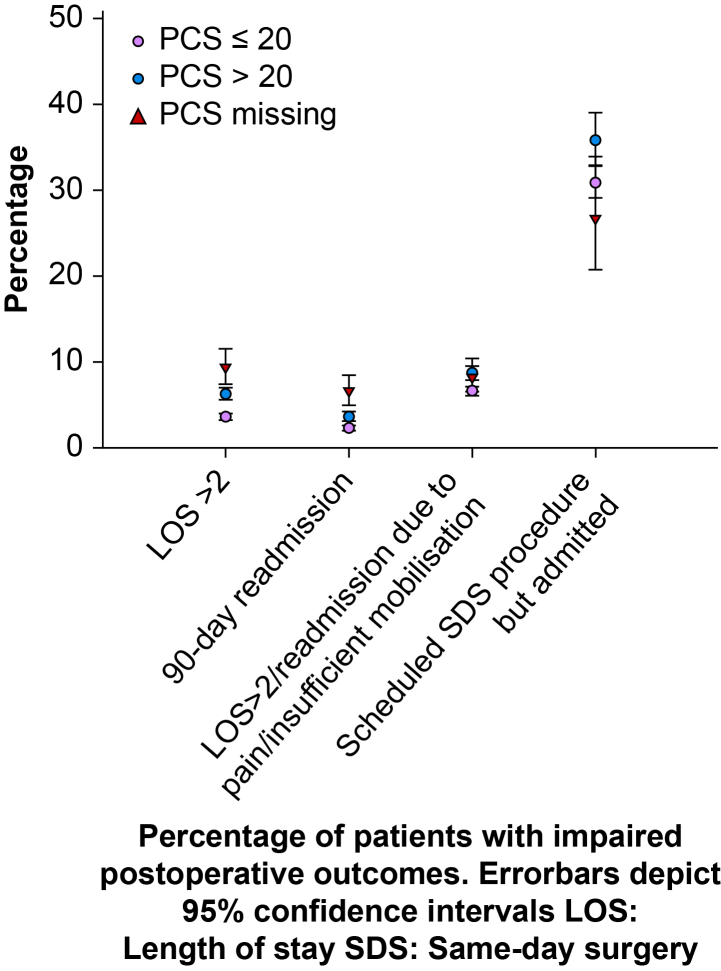
Percentage of patients with impaired postoperative outcomes. Errorbars depict 95% confidence intervals PCS: pain catastrophizing scale LOS: Length of stay SDS: Same-day surgery.

The final cohort consisted of 15 695 procedures in 13 782 individual patients (Fig. 1), having surgery between 9 September 2022 and 6 June 2025. Of these, 5225 had a PCS >20, with a median of 29 (IQR: 24–35), and 9573 had a PCS of ≤ 20, with a median of 10 (IQR: 4–15). In 897 (6%) patients, there were no data on PCS, primarily due to patients opting out of receiving questionnaires. Most patient characteristics were similar between PCS >20 and PCS ≤ 20, except for sex, use of walking aids, and preoperative opioid and psychotropic treatment (Table 1). Also, fewer patients with PCS >20 were found eligible for same-day surgery. Overall median LOS was 1 day (IQR: 0–1), with 5213 (35%) patients included in same-day surgical pathways. There were 1080 (7.3%, CI: 6.9-7.7) readmissions, and 35 (0.2%, CI: 0.2–0.3) patients died within 90 days of surgery. Finally, there were 409 (2.8%, CI: 2.5–3.0) cases with a LOS >2 or readmissions due to pain or insufficient mobilisation. The number of patients with a LOS >2 days was 341 (3.6%, CI: 3.2–4.0) in those with PCS ≤20 and 329 (6.3%, CI: 5.6–7.0) in those with PCS >20 (Fig. 2). After adjusting for confounders, PCS >20 was significantly associated with LOS >2 days (odds ratio [OR]: 1.54; CI: 1.29–1.83; *P*<0.001) and all-cause 90-day readmissions (2.3%; CI: 2.0–2.6 vs 3.6%, CI: 3.1–4.2; OR: 1.18; CI: 1.03–1.35; *P*=0.020). PCS >20 was also associated with the composite outcome of LOS >2 or 90-day readmissions due to pain or insufficient mobilisation (6.6%, CI: 6.1–7.1 vs 8.7%, CI: 7.9–9.5; OR: 1.38; CI: 1.11–1.72; *P*=0.004). Finally, PCS >20 was associated with admission to hospital despite planned same-day surgical procedures (30.9%, CI: 29.1–32.8 vs 35.8%; CI: 32.9–39.0; OR: 1.22; CI: 1.06–1.40; *P*=0.005) (Fig. 2 and Table 2). The results on the remaining included variables can be found in Supplementary Table 1.

**Table 1:** Patient characteristics. Numbers are provided as *n* (%) unless otherwise noted. IQR, inter-quartile range; PCS, pain catastrophising scale; THA, total hip arthroplasty; TKA, total knee arthroplasty; UKA, unicompartmental knee arthroplasty ∗Defined as a haemoglobin of <13 g dl^−1^ regardless of gender.

Characteristic	PCS >20 (*n=*5225)	PCS ≤ 20 (*n=*9573)	Missing PCS (*n=*897)
Age median (range)	70 (20–93)	71 (19–96)	72 (21–93)
Females	3419 (65)	5236 (55)	538 (60.0)
PCS median (IQR)	29 (24–35)	10 (4–15)	-
Consent to chart review only	159 (3)	159 (2)	507 (57)
Cohabitating	3343 (64)	6638 (69)	435 (49)
missing data	57 (1)	97 (1)	206 (23)
Clinical Frailty Scale median (IQR)	3 (2–4)	3 (2–3)	3 (2–4)
missing data	6 (0)	13 (0)	2 (0)
Use of walking aids	1855 (36)	1998 (21)	222 (25)
missing data	66 (1)	93 (1)	226 (25.2)
Opioid use	896 (17)	869 (9)	134 (15)
missing data	2 (0)	10 (0)	0 (0)
Cardiac medication	3239 (62)	5652 (59)	538 (60)
missing data	3 (0)	6 (0)	0 (0)
Pulmonary medication	755 (14)	1134 (12)	124 (14)
missing data	23 (0)	33 (0)	2 (0)
Psychotropic treatment	756 (15)	862 (9)	115 (13)
missing data	16 (0)	21 (0)	3 (0)
Hyperglycaemic treatment	663 (13)	956 (10)	119 (13)
missing data	119 (2.3)	277 (2.9)	5 (1)
Anaemia∗	1304 (25)	1932 (20)	224 (25)
missing data	4 (0)	3 (0)	0 (0)
Procedure THA TKA UKA	2377 (46) 1902 (36) 946 (18)	3944 (41) 3773 (40) 1846 (19)	398 (45) 317 (35) 182 (20)
Eligible for same-day surgery	1833 (35)	4430 (46)	309 (34)
missing data	3 (0)	2 (0)	1 (0)
Planned for same-day surgery	1518 (29)	3695 (39)	251 (28)
missing data	15 (0)	46 (1)	2 (0)

**Table 2:** Adjusted odds ratios between the pain catastrophising scale (PCS) >20 compared to PCS ≤20 for the study outcomes. In total, 715 (4.8%) patients from the total cohort were excluded from logistic regression analysis due to missing data in specific variables. LOS, length of hospital stay; THA, total hip arthroplasty; TKA, total knee arthroplasty; UKA, unicompartmental knee arthroplasty. ∗ The analysis of patients having planned same-day surgery included 5009 patients with 2014 THA, 1705 TKA, and 1290 UKA.

Population	LOS >2, including pain and insufficient mobilisation		Ninety-day readmissions, including pain and insufficient mobilisation		LOS >2 or 90-day readmissions due to pain/insufficient mobilisation		Overnight admission in planned same-day surgery ∗	
	Adjusted odds ratio	*P*-value	Adjusted odds ratio	*P*-value	Adjusted odds ratio	*P*-value	Adjusted odds ratio	*P*-value
Total (*n*:14 083)	1.54 (1.29–1.83)	<0.001	1.18 (1.03–1.35)	0.020	1.38 (1.11–1.72)	0.004	1.22 (1.06–1.40)	0.005
THA (*n*:6015)	1.56 (1.20–2.03)	0.001	1.23 (1.00–1.52)	0.049	1.22 (0.85–1.76)	0.287	1.30 (1.06–1.59)	0.013
TKA (*n*:5381)	1.74 (1.36–2.23)	<0.001	1.02 (0.82–1.26)	0.890	1.70 (1.27–2.28)	<0.001	1.18 (0.93–1.49)	0.180
UKA (*n*:2687)	0.59 (0.30–1.17)	0.129	1.51 (1.08–2.11)	0.015	0.86 (0.55–1.34)	0.491	1.10 (0.81–1.59)	0.551

### Sensitivity analyses

The procedure-specific analysis found that the strength of associations varied depending on the type of procedure. Thus, the relation between PCS >20 and admission despite planned same-day discharge was significant only in THA. In contrast, the only significant association for PCS >20 in UKA was an increased risk of 90-day readmissions (Table 2).

In the 897 patients with missing data on PCS, the median LOS was also 1 day (IQR: 1–1) and 83 (9.3%; CI: 7.4–11.5) patients had a LOS >2 days. Within 90 days after surgery, 74 (8.2%; CI: 6.5–10.4) patients were readmitted, and 3 (0.3%; CI: 0.1–1.0) died. LOS >2 days or readmissions due to pain or insufficient mobilisation occurred in 59 (6.6%; CI: 5.7–9.4). Of the 251 patients with planned same-day surgery, 67 (26.1%; CI: 20.7–33.9) were admitted to hospital (Fig. 2).

When testing the robustness of the association between PCS >20 and our primary outcome of a LOS of >2 days, we found that this would remain significant regardless of the presence or distribution of an unmeasured confounder with an OR of 1.10. The point estimate of the OR for PCS >20 would also be at least 1.40 regardless of the distribution of the confounder in those with and without PCS >20, respectively (Supplementary Table 2). However, an unmeasured confounder influencing the OR of the primary outcome by 2.00 could make the association insignificant if present in 30% of those with PCS >20 and in none with PCS ≤ 20 (Supplementary Table 3).

## Discussion

We report a significant association between the PCS and an increased risk of impaired postoperative outcomes after hip and knee arthroplasty within an optimised fast-track setting, including within a specific same-day surgical pathway. Furthermore, PCS was associated with both an increased 90-day readmission rate and increased LOS >2 days or 90-day readmission specifically due to pain or insufficient mobilisation, and with not being discharged on the day of surgery in planned same-day surgical procedures.

Our results are important, considering that there may be an effect on postoperative pain after TKA when increasing single-dose dexamethasone to 1 mg kg^−1^, specifically in patients with a PCS >20,^13^ instead of using the 24 mg fixed dose currently standard within the participating centres.^17^ However, only one of the participating centres applied this in clinical practice during the study period, and whether it positively affects LOS and readmission rate in these patients remains to be investigated. Our results also imply that targeting patients with a PCS >20 for future interventional pain studies, e.g. repeated peripheral nerve blocks or novel adjuvant analgesic techniques, and including prolonged LOS or readmissions as outcomes, is clinically relevant, as about one third of all patients have a PCS of >20. Thus, our study supports the concept of a stratified inclusion of patients in interventional studies by choosing those who are most likely to experience increased postoperative pain.^24^ In this context, the ideal threshold for PCS stratification remains debatable but is most likely somewhere between 20 and 30.^7^ The procedure-specific analysis revealed that the influence of PCS >20 was not the same in THA, TKA and UKA. This illustrates that patients scheduled for THA, TKA and UKA may be considered as different populations and not simply as patients undergoing arthroplasty. In THA, an association between PCS >20 and LOS>2 days, 90-day readmission rate, and admission despite planned same-day surgery, but not regarding specific pain- or mobilisation-related causes for having LOS >2 days or 90-day readmissions, was found. In contrast, in TKA, the risk of LOS >2 days or readmission due to pain or insufficient mobilisation increased, but with no increased risk of 90-day readmissions or admission despite planned same-day surgery. Finally, in UKA, the influence of PCS >20 was mostly attenuated, as only the risk of an increased 90-day readmission rate was increased. The reason for these differences is likely multifactorial and influenced by differences in postoperative pain intensity, with patients undergoing TKA being at risk of increased immediate severe postoperative pain for a prolonged duration compared to those undergoing THA and UKA. In contrast, patients undergoing THA require fewer opioids but are often elderly and might have difficulties mobilising caused by reasons other than pain, e.g. postoperative orthostatic intolerance.^25^ Interestingly, despite UKA being considered a less comprehensive surgical procedure and with a higher likelihood of same-day discharge,^5^ our data revealed a considerable increase in the risk of readmissions within 90 days in patients with a PCS >20. However, the results of the procedure-specific analyses must be interpreted with care, as our study was not powered to evaluate procedure-specific outcomes.

Study limitations include differences in analgesic treatment between the participating departments, most importantly regarding the use of increased doses of dexamethasone and peripheral nerve blocks in TKA. Thus, although we have previously found that peripheral nerve blocks do not influence LOS in fast-track TKA,^19^ whether they may have a positive effect specifically in those with PCS >20 is not known. Also, the results in the cohort of patients with missing PCS data imply that a considerable number of these patients may have a PCS >20, as the distribution of patient characteristics and postoperative outcomes was often similar to those with PCS >20. However, it must be considered that the fraction of patients scheduled for same-day surgery who were admitted was lowest among those with missing PCS data. It could be argued that we should have used multiple imputation to address the issue of missing data, but we refrained from this as it was <5%. Furthermore, given that the main reason for missing PCS data was patients opting out of receiving questionnaires, it seems unjustified to assume that data on PCS were missing at random or missing completely at random.^26^ We may also have conducted further subgroup analyses, e.g. on those with preoperative opioids or those using psychotropic medication, as there could be interactions with PCS. However, the indication for psychotropic medication in most Danish patients undergoing arthroplasty is due to minor depression,^27^ and the pre-study directed acyclic graphs did not reveal a major issue when including these patients. Finally, it could be argued that we should have adjusted for multiple comparisons regarding the level of significance for the secondary outcomes. However, especially the procedure-specific analysis needs to be interpreted with caution, regardless of any such adjustments, as the study was only powered for the primary outcome.

Study strengths include the inclusion of similar fast-track protocols, assuring that the main perioperative care was alike between departments. Also, the inclusion of preoperative prospective data and meticulous follow-up allows for a qualified analysis while adjusting for relevant confounders. In this context, PCS would remain significantly associated with the primary outcome of a LOS >2 days despite the presence of an unmeasured confounder. Finally, the multicentre collaboration, encompassing about 40% of all annual procedures in Denmark, improves the generalisability of our results.

In conclusion, a PCS >20 was associated with increased risk of LOS >2 days an increased 90-day all-cause readmission rate after fast-track hip and knee arthroplasty. Furthermore, PCS was also associated with LOS >2 days or 90-day readmissions, specifically due to pain or mobilisation issues and admission in procedures scheduled for same-day discharge. Future interventional studies could consider patients with PCS >20, as these individuals may benefit the most from targeted pain interventions.

## Authors’ contributions

Study conception and design: CJ, SK, HK, CV

Construction of the data-collection instruments and implementing them at their institutions: all authors.

The ERAS protocols at their institutions: ML, KG, CV, MB, SO, TBH, MA

Writing the statistical analysis plan and conducting the primary data analysis: CJ with input from SK and CV

Drafting the manuscript: CJ

Manuscript revision: SK, CV

Final manuscript and access to the data: all authors.

## Funding

Novo Nordisk Foundation, Denmark (grant number: NNF21SA0073760) and the Candys Foundation, Denmark.

## Declarations of interest

CJ is a member of the board of the Danish Society of Ambulatory Surgery and has received personal speaker honoraria and reimbursement of travel expenses from Pharmacosmos, Denmark. CV received travel expenses from Stryker, paid to his institution, with no relevance to the present study. SO is Editor in Chief of Acta Orthopaedica and has received speaker’s fees from Heraeus. The remaining authors have no conflicts to declare.
