# Supplementary material for: Pain catastrophising as a risk factor for hospitalisation and readmissions in fast-track hip and knee arthroplasty, an observational multicentre cohort study
Source: BJA Open. 2026 Jun 1;18:100564. doi: 10.1016/j.bjao.2026.100564 (PMC13251635; doi:10.1016/j.bjao.2026.100564)
Supplement: Multimedia component 1 [file mmc1.docx]

Supplementary Table 1 Adjusted odds ratios of the remaining variables for all study outcomes

|  | LOS >2 including pain and insufficient mobilisation | | 90-days readmissions including pain and insufficient mobilisation | | LOS >2 or 90-days readmissions due to pain/insufficient mobilisation only | | Overnight admission in planned same-day surgery^2^ | |
| --- | --- | --- | --- | --- | --- | --- | --- | --- |
|  | Adjusted Odd Ratio | P-value | Adjusted Odd Ratio | P-value | Adjusted Odd Ratio | P-value | Adjusted Odd Ratio | P-value |
| Total cohort (n:14,083) | |  |  |  |  |  |  |  |
| Male gender | 1.54 (1.29-1.83) | <0.001 | 1.53 (1.33-1.76) | <0.001 | 0.76 (0.60-0.98) | 0.031 | 0.62 (0.55-0.71) | <0.001 |
| Age/year | 1.01 (1.0-1.02) | 0.045 | 1.02 (1.01-1.03) | <0.001 | 1.00 (0.99-1.02) | 0.633 | 1.01 (1.00-1.01) | 0.074 |
| CFS | 1.34 (1.23-1.46) | <0.001 | 1.27 (1.18-1.36) | <0.001 | 1.30 (1.16-1.45) | <0.001 | 1.10 (1.01-1.21) | 0.037 |
| Living alone | 1.37 (1.15-1.63) | <0.001 | 1.11 (0.96-1.28) | 0.159 | 1.50 (1.20-1.87) | <0.001 | 1.04 (0.87-1.26) | 0.659 |
| Antihyperglycemic treatment | 1.13 (0.88-1.44) | 0.347 | 1.18 (0.98-1.41) | 0.086 | 1.26 (0.94-1.69) | 0.124 | 0.98 (0.76-1.27) | 0.885 |
| Treatment for cardiac disease | 0.96 (0.79-1.16) | 0.651 | 1.21 (1.04-1.40) | 0.015 | 1.02 (0.80-1.30) | 0.873 | 1.00 (0.88-1.15) | 0.980 |
| Treatment for pulmonary disease | 1.08 (0.858-1.35) | 0.522 | 1.21 1.01-1.44) | 0.035 | 1.11 (0.84-1.47) | 0.477 | 0.92 (0.74-1.14) | 0.431 |
| Psychotropic treatment | 1.45 (1.15-1.82) | 0.001 | 1.33 (1.24-1.61) | 0.003 | 1.26 (9.43-1.69) | 0.118 | 1.10 (0.87-1.38) | 0.439 |
| Opioid use | 1.25 (0.99-1.55) | 0.052 | 1.25 (1.05-1.49) | 0.014 | 1.59 (1.22-2.07) | <0.001 | 1.13 (0.89-1.43) | 0.322 |
| Anaemia^1^ | 1.39 (1.16-1.67) | <0.001 | 1.44 (1.24-1.67 | <0.001 | 1.18 (0.93-1.49) | 0.167 | 1.16 (0.97-1.38) | 0.108 |
| Planned same-day discharge | 0.27 (0.20-0.37) | <0.001 | 0.79 (0.66-0.95) | 0.011 | 0.35 (0.24-0.51) | <0.001 | - | - |
| TKA | 1.57 (1.31-1.87) | <0.001 | 1.22 (1.06-1.41) | 0.007 | 2.04 (1.62-2.57) | <0.001 | 1.11 (0.96-1.28) | 0.165 |
| UKA | 0.66 (0.47-0.91) | 0.011 | 1.02 (0.84-1.24) | 0.812 | 0.91 (0.61-1.36) | 0.646 | 0.54 (0.46-0.64) | <0.001 |
| THA only (n:6015) |  |  |  |  |  |  |  |  |
| Male gender | 0.54 (0.38-0.75) | <0.001 | 1.58 (1.27-1.96) | 0.05 | 0.62 (0.40-0.97) | 0.036 | 0.62 (0.51-0.76) | <0.001 |
| Age/year | 1.02 (1.01-1.04) | 0.005 | 1.03 (1.02-1.04) | <0.001 | 1.02 (1.00-1.04) | 0.066 | 1.00 (0.99-1.01) | 0.635 |
| CFS | 1.20 (1.05-1.36) | 0.006 | 1.20 (1.09-1.33) | <0.001 | 1.17 (0.98-1.39) | 0.093 | 1.13 (0.98-1.30) | 0.085 |
| Living alone | 1.65 (1.26-3.17) | <0.001 | 1.00 (0.80-1.24) | 0.965 | 1.59 (1.09-2.32) | 0.017 | 1.14 (0.86-1.52) | 0.358 |
| Antihyperglycemic treatment | 1.03 (0.67-1.59) | 0.884 | 1.43 (0.92-1.46) | 0.018 | 1.11 (0.62-2.00) | 0.723 | 1.55 (1.01-2.39) | 0.046 |
| Treatment for cardiac disease | 0.85 (0.64-1.13) | 0.270 | 1.16 (0.92-1.46) | 0.208 | 0.94 (0.63-1.39) | 0.723 | 0.95 (0.77-1.17) | 0.620 |
| Treatment for pulmonary disease | 1.09 (0.77-1.55) | 0.630 | 1.10 (0.84-1.45) | 0.492 | 0.86 (0.51-1.44) | 0.564 | 1.40 (0.98-1.86) | 0.620 |
| Psychotropic treatment | 1.51 (1.06-2.14) | 0.023 | 1.64 (1.23-2.18) | <0.001 | 1.36 (0.82-2.24) | 0.236 | 0.88 (0.61-1.26) | 0.475 |
| Opioid use | 1.39 (1.01-1.92) | 0.043 | 1.33 (1.03-1.72) | 0.031 | 1.80 (1.17-2.76) | 0.008 | 1.23 (0.85-1.78) | 0.274 |
| Anaemia | 1.47 (1.12-1.93) | 0.006 | 1.20 (0.95-1.51) | 0.122 | 1.00 (0.68-1.47) | 0.991 | 1.20 (0.92-1.56) | 0.188 |
| Planned same-day discharge | 0.33 (0.20-0.55) | <0.001 | 0.76 (0.57-1.02) | 0.064 | 0.39 (0.21-0.74) | 0.004 | - | - |
| TKA (n:5381) |  |  |  |  |  |  |  |  |
| Male gender | 0.73 (0.55-0.97) | <0.001 | 1.50 (1.20-1.86) | <0.001 | 0.88 (0.63-1.22) | 0.431 | 0.61 (0.49-0.77) | <0.001 |
| Age/year | 1.0 (0.99-1.01) | 0.969 | 1.01 (1.20-1.86) | 0.038 | 1.00 (0.98-1.02) | 0.895 | 1.01 (0.99-1.02) | 0.357 |
| CFS | 1.415 (1.27-1.65) | <0.001 | 1.33 (1.19-1.48) | <0.001 | 1.35 (1.16-1.58) | <0.001 | 1.06 (0.90-1.24) | 0.497 |
| Living alone | 1.26 (0.98-1.62) | 0.070 | 1.17 (0.94-1.45) | 0.170 | 1.53 (1.14-2.05) | 0.005 | 1.21 (0.88-1.66) | 0.232 |
| Antihyperglycemic treatment | 1.02 (0.73-1.43) | 0.897 | 0.95 (0.71-1.26) | 0.700 | 1.16 (0.80-1.69) | 0.443 | 0.55 (0.35-0.84) | 0.007 |
| Treatment for cardiac disease | 1.08 (0.82-1.42) | 0.595 | 1.15 (0.91-1.46) | 0.244 | 1.10 (0.79-1.53) | 0.569 | 0.96 (0.77-1.20) | 0.737 |
| Treatment for pulmonary disease | 0.99 (0.71-1.39) | 0.950 | 1.36 (1.04-1.79) | 0.025 | 1.17 (0.80-1.71) | 0.426 | 0.64 (0.44-0.94) | 0.022 |
| Psychotropic treatment | 1.39 (1.01-1.92) | 0.047 | 1.21 (0.91-1.63) | 0.197 | 1.19 (0.81-1.76) | 0.379 | 1.45 (0.99-2.13) | 0.058 |
| Opioid use | 1.11 (0.80-1.54) | 0.542 | 1.34 (1.02-1.77) | 0.037 | 1.46 (1.01-2.10) | 0.044 | 1.15 (0.78-1.69) | 0.483 |
| Anaemia | 1.38 (1.07-1.79) | 0.015 | 1.74 (1.39-2.78) | <0.001 | 1.35 (0.99-1.83) | 0.057 | 1.22 (0.91-1.65) | 0.185 |
| Planned same-day discharge | 0.26 (0.17-0.42) | <0.001 | 0.71 (0.53-0.96) | 0.025 | 0.31 (0.18-0.52) | <0.001 | - | - |
| UKA (n:2687) |  |  |  |  |  |  |  |  |
| Male gender | 0.71 (0.37-1.35) | 0.289 | 1.59 (1.12-2.24) | 0.009 | 0.96 (0.62-1.48) | 0.853 | 1.01 (0.52-1.96) | 0.978 |
| Age/year | 0.99 (0.96-1.03) | 0.719 | 1.02 (1.00-1.04) | 0.096 | 0.99 (0.97-1.01) | 0.430 | 1.00 (0.96-1.04) | 0.970 |
| CFS | 1.47 (1.08-2.00) | 0.014 | 1.30 (1.09-1.56) | 0.004 | 1.12 (0.89-1.41) | 0.346 | 1.06 (0.68-1.65) | 0.808 |
| Living alone | 0.84 (0.44-1.60) | 0.599 | 1.29 (0.89-1.04) | 0.096 | 1.07 (0.66-1.74) | 0.776 | 0.91 (0.32-2.59) | 0.859 |
| Antihyperglycemic treatment | 2.17 (1.04-4.51) | 0.014 | 1.23 (0.79-1.91) | 0.353 | 1.28 (0.70-2.33) | 0.422 | 1.20 (0.37-3.87) | 0.760 |
| Treatment for cardiac disease | 0.85 (0.42-1.72) | 0.652 | 1.45 (1.00-2.13) | 0.053 | 0.98 (0.62-1.53) | 0.911 | 083 (0.42-1.63) | 0.590 |
| Treated pulmonary disease | 1.37 (0.66-2.84) | 0.391 | 1.19 (0.77-1.84) | 0.446 | 1.20 (0.68-2.10) | 0.534 | 1.07 (0.38-3.00) | 0.894 |
| Psychotropic treatment | 1.27 (0.55-2.89) | 0.578 | 0.98 (0.59-1.64) | 0.946 | 1.07 (0.56-2.03) | 0.197 | 0.91 (0.27-3.02) | 0.877 |
| Opioid use | 1.22 (0.52-2.88) | 0.644 | 0.77 (0.43-1.37) | 0.370 | 1.25 (0.64-2.42) | 0.510 | 1.20 (0.35-4.14) | 0.771 |
| Anaemia | 0.88 (0.41-1.90) | 0.748 | 1.39 (0.92-2.09) | 0.119 | 0.97 (0.55-1.44) | 0.911 | 0.90 (0.32-2.54) | 0.842 |
| Planned same-day discharge | 0.16 (0.06-0.44) | <0.001 | 0.98 (0.66-1.46) | 0.938 | 0.89 (0.56-1.44) | 0.643 | - | - |
| In total 715 (4.8%) patients from the total cohort were excluded from logistic regression analysis due to missing data in specific variables. LOS: Length of hospital stay THA: total hip arthroplasty TKA: total knee arthroplasty UKA: unicompartmental knee arthroplasty  ^1^ Defined as a haemoglobin < 13g/dL regardless of gender ^2^The analysis of patients having planned same-day surgery included 5009 patients with 2014 THA, 1705 TKA and 1290 UKA. | | | | | | | | |

Supplementary Table 2. Sensitivity analysis on the robustness of the association between PCS >20 and LOS > 2 days, including an unmeasured confounder with an OR of 1.10

|  | Incidence of unmeasured confounder in patients with PCS ≤20 | | | | | | |
| --- | --- | --- | --- | --- | --- | --- | --- |
|  |  | 0 | 0.1 | 0.2 | 0.3 | 0.4 | 0.5 |
| Incidence of unmeasured confounder in patients with PCS >20 | 0 | 1.54 (1.29-1.83) | 1.56 (1.30-1.85) | 1.57 (1.32-1.87) | 1.59 (1.33-1.88) | 1.60 (1.34-1.90) | 1.62 (1.35-1.92) |
|  | 0.1 | 1.52 (1.28-1.81) | 1.54 (1.29-1.83) | 1.56 (1.30-1.85) | 1.57 (1.32-1.87) | 1.59 (1.33-1.88) | 1.60 (1.34-1.90) |
|  | 0.2 | 1.51 (1.26-1.79) | 1.52 (1.28-1.81) | 1.54 (1.29-1.83) | 1.56 (1.30-1.85) | 1.57 (1.32-1.87) | 1.59 (1.33-1.88) |
|  | 0.3 | 1.50 (1.25-1.78) | 1.51 (1.27-1.79) | 1.53 (1.28-1.81) | 1.54 (1.29-1.83) | 1.55 (1.30-1.85) | 1.57 (1.32-1.87) |
|  | 0.4 | 1.48 (1.24-1.76) | 1.50 (1.25-1.78) | 1.51 (1.27-1.79) | 1.53 (1.28-1.81) | 1.54 (1.29-1.83) | 1.55 (1.30-1.85) |
|  | 0.5 | 1.47 (1.23-1.74) | 1.48 (1.24-1.76) | 1.50 (1.25-1.78) | 1.51 (1.27-1.80) | 1.53 (1.28-1.81) | 1.54 (1.29-1.83) |
|  | 0.6 | 1.45 (1.22-1.73) | 1.47 (1.23-1.74) | 1.48 (1.24-1.76) | 1.50 (1.25-1.78) | 1.51 (1.27-1.78) | 1.53 (1.28-1.81) |
|  | 0.7 | 1.44 (1.21-1.71) | 1.45 (1.22-1.73) | 1.47 (1.23-1.74) | 1.48 (1.24-1.76) | 1.50 (1.25-1.76) | 1.51 (1.27-1.80) |
|  | 0.8 | 1.43 (1.19-1.69) | 1.44 (1.21-1.71) | 1.45 (1.22-1.73) | 1.47 (1.23-1.75) | 1.48 (1.24-1.75) | 1.50 (1.25-1.78) |
|  | 0.9 | 1.41 (1.18-1.68) | 1.43 (1.20-1.70) | 1.44 (1.21-1.71) | 1.46 (1.22-1.73) | 1.47 (1.23-1.73) | 1.48 (1.24-1.76) |
|  | 1 | 1.40 (1.17-1.66) | 1.41 (1.18-1.68) | 1.43 (1.20-1.70) | 1.44 (1.21-1.71) | 1.46 (1.22-1.71) | 1.47 (1.23-1.75) |
|  | | Changes to the OR (95%CI) of PCS >20 with changes in incidence of an unmeasured confounder with an OR of 1.10 for LOS >2 days. | | | | | |

|  | Incidence of unmeasured confounder in patients with PCS ≤ 20 | | | | | | | |
| --- | --- | --- | --- | --- | --- | --- | --- | --- |
|  |  | 0 | | 0.1 | 0.2 | 0.3 | 0.4 | 0.5 |
| Incidence of unmeasured confounder in patients with PCS > 20 | 0 | 1.54 (1.29-1.83) | | 1.69 (1.42-2.01) | 1.85 (1.55-2.20) | 2.00 (1.68-2.38) | 2.16 (1.81-2.56) | 2.31 (1.94-2.75) |
|  | 0.1 | 1.40 (1.17-1.66) | | 1.54 (1.29-1.83) | 1.68 (1.41-2.00) | 1.82 (1.52-2.16) | 1.96 (1.64-2.33) | 2.10 (1.76-2.50) |
|  | 0.2 | 1.28 (1.08-1.53) | | 1.41 (1.18-1.68) | 1.54 (1.29-1.83) | 1.67 (1.40-1.98) | 1.80 (1.51-2.14) | 1.93 (1.61-2.29) |
|  | 0.3 | 1.18 (0.99-1.41) | | 1.30 (1.09-1.55) | 1.42 (1.19-1.69) | 1.54 (1.29-1.83) | 1.66 (1.39-1.97) | 1.78 (1.49-2.11) |
|  | 0.4 | 1.10 (0.92-1.31) | | 1.21 (1.01-1.44) | 1.32 (1.11-1.57) | 1.43 (1.20-1.70) | 1.54 (1.29-1.83) | 1.65 (1.38-1.96) |
|  | 0.5 | 1.03 (0.86-1.22) | | 1.13 (0.95-1.34) | 1.23 (1.03-1.46) | 1.33 (1.12-1.59) | 1.44 (1.20-1.71) | 1.54 (1.29-1.83) |
|  | 0.6 | 0.96 (0.81-1.14) | | 1.06 (0.89-1.26) | 1.16 (0.97-1.37) | 1.25 (1.05-1.49) | 1.35 (1.13-1.60) | 1.44 (1.21-1.72) |
|  | 0.7 | 0.91 (0.76-1.08) | | 1.00 (0.83-1.18) | 1.09 (0.91-1.29) | 1.18 (0.99-1.40) | 1.27 (1.06-1.51) | 1.36 (1.14-1.61) |
|  | 0.8 | 0.86 (0.72-1.02) | | 0.94 (0.79-1.12) | 1.03 (0.86-1.22) | 1.11 (0.93-1.32) | 1.20 (1.00-1.42) | 1.28 (1.08-1.53) |
|  | 0.9 | 0.81 (0.68-0.96) | | 0.89 (0.75-1.06) | 0.97 (0.81-1.16) | 1.05 (0.88-1.25) | 1.13 (0.95-1.35) | 1.22 (1.02-1.44) |
|  | 1 | 0.77 (0.65-0.92) | | 0.42 (0.35-1.01) | 0.92 (0.77-1.10) | 1.00 (0.84-1.19) | 1.08 (0.90-1.28) | 1.16 (0.97-1.37) |
|  | | | Changes to the OR (95%CI) of PCS >20 with changes in incidence of an unmeasured confounder with an OR of 2.00 for LOS >2 days. Black line indicates threshold for insignificance. | | | | | |

Supplementary Table 3. Sensitivity analysis on the robustness of the association between PCS >20 and LOS > 2 days, including an unmeasured confounder with an OR of 2
