# Supplementary material for: Pain catastrophising as a risk factor for hospitalisation and readmissions in fast-track hip and knee arthroplasty, an observational multicentre cohort study
Source: BJA Open. 2026 Jun 1;18:100564. doi: 10.1016/j.bjao.2026.100564 (PMC13251635; doi:10.1016/j.bjao.2026.100564)
Supplement: Multimedia component 2 [file mmc2.docx]

**Study analysis plan: Outcomes in patients with high pain catastrophizing scores after fast-track hip and knee arthroplasty, a prospective cohort study.**

Setting: 8 Danish high-volume arthroplasty departments participating in the Fast-track Center for Hip and Knee Replacement collaboration and database. (<https://fast-track.health/>)

Data is extracted from the Fast-track Center database registered on ClinicalTrials.gov. including all primary unilateral procedures with completed 90-days follow-up and completed preoperative medicine status. In one department which started registration of PCS on 15.12.2023 included procedures will be from this data and onwards. Patients refusing to consent to participation are excluded from analysis.

Primary outcome: Association between PCS score of >20 and LOS >2 days

Secondary outcomes: 90-days readmissions with overnight stay, composite of LOS >2 days or 90-days readmission related to pain or mobilization, Successful discharge on day of surgery in patients included in the same-day surgical pathway

Statistics: Logistic regression adjusting for patient and procedure related potential confounders. Relevant confounders will be based on existing literature and clinical experience and using directed acyclic graphs (DAG’s).

CODE for DAG using https://www.dagitty.net/dags.html#:

dag {

bb="-4.548,-4.358,5.031,5.152"

"Anaemia (<13g/dL" [adjusted,pos="1.147,2.325"]

"Cardiac medication" [adjusted,pos="-3.750,-1.240"]

"LOS>2" [outcome,pos="-0.029,0.151"]

"Sameday surgery" [adjusted,pos="-0.194,-3.565"]

"preop opioids" [adjusted,pos="-3.084,2.653"]

"psychiatric drugs" [adjusted,pos="-3.369,0.359"]

"pulmonary drugs" [adjusted,pos="-2.563,-3.292"]

"unmeasured conditions" [latent,pos="3.550,-2.023"]

"walking aids" [adjusted,pos="1.381,-3.314"]

CFS [adjusted,pos="-1.305,-1.803"]

PCS [exposure,pos="-1.064,3.307"]

age [adjusted,pos="1.641,-1.408"]

cohabitation [adjusted,pos="2.769,1.359"]

diabetes [adjusted,pos="-3.241,1.439"]

gender [adjusted,pos="0.914,3.895"]

procedure [adjusted,pos="-0.006,4.359"]

"Anaemia (<13g/dL" -> "LOS>2"

"Cardiac medication" -> "LOS>2"

"Cardiac medication" -> CFS

"Sameday surgery" -> "LOS>2"

"Sameday surgery" <-> age

"preop opioids" -> "LOS>2"

"preop opioids" -> PCS

"psychiatric drugs" -> "LOS>2"

"psychiatric drugs" -> CFS

"pulmonary drugs" -> "LOS>2"

"pulmonary drugs" -> CFS

"unmeasured conditions" -> "LOS>2"

"walking aids" -> "LOS>2"

"walking aids" -> "Sameday surgery"

"walking aids" -> CFS

"walking aids" -> age

CFS -> "LOS>2"

CFS -> "Sameday surgery"

PCS -> "LOS>2"

age -> "Anaemia (<13g/dL"

age -> "LOS>2"

age -> CFS

cohabitation -> "LOS>2"

cohabitation -> age

diabetes -> "LOS>2"

gender -> "Anaemia (<13g/dL"

gender -> "LOS>2"

procedure -> "LOS>2"

}
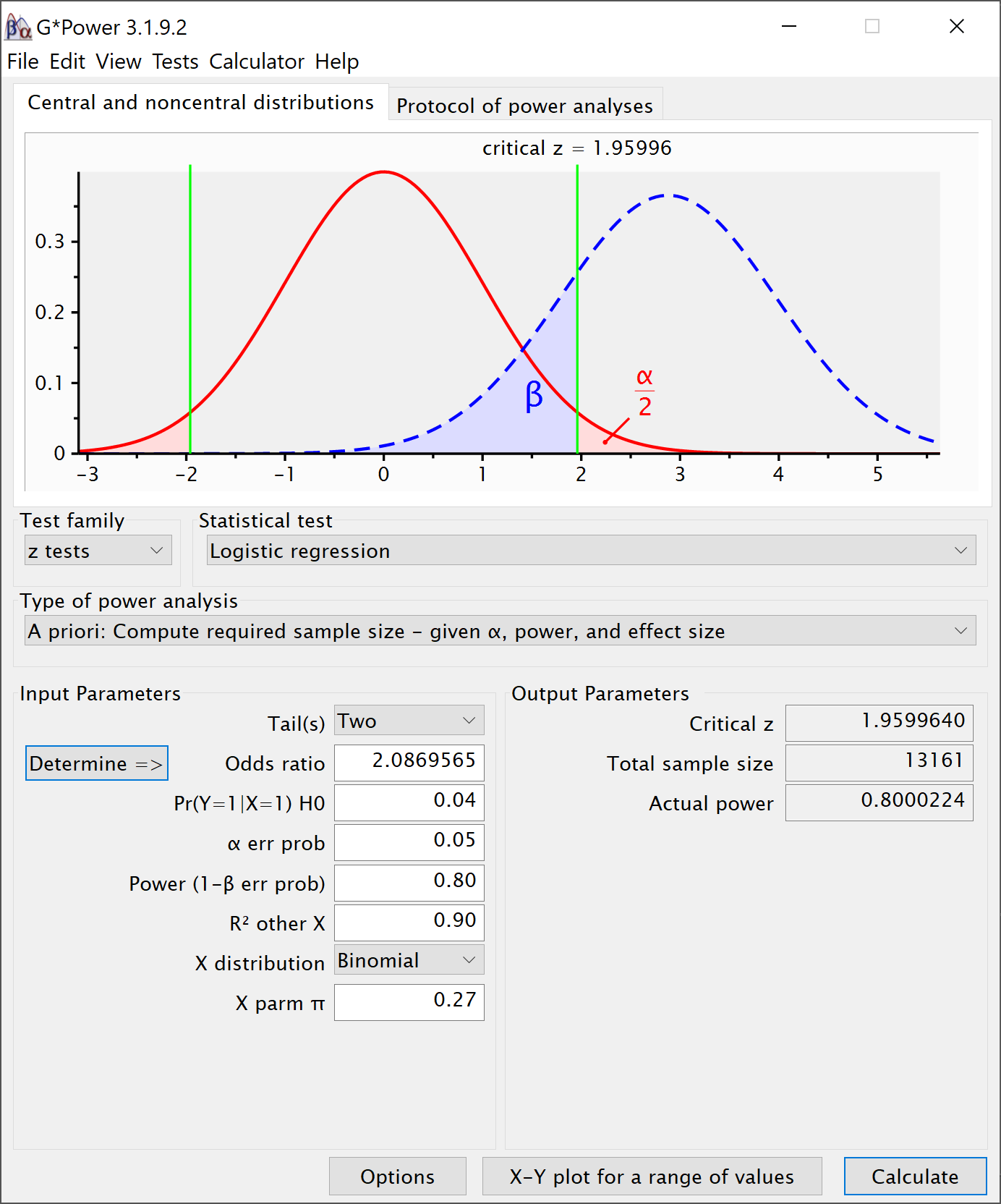


Power considerations: Assuming that PCS contributes 10% to the primary outcome and based on an existing dataset with 4% of patients having a LOS >2 days and an incidence of PCS >20 of 27% the following pre-study power analysis was performed using Gpower:

Handling of missing data:
Patients with missing data for variables included in the regression analysis will be excluded. Patients with missing PCS data will be included as a “missing PCS” group” and be reported separately.

Sensitivity analysis: Procedure specific analysis will be conducted on the primary for outcome for THA, TKA and UKA separately in order to investigate the procedure specific robustness of the results. A sensitivity analysis on potential imbalanced unmeasured confounding for an unmeasured confounder with an odds ratio of 1.1 and 2.00 will also be conducted to investigate the potential impact of unmeasured confounding.^1^

1. Lin DY, Psaty BM, Kronmal RA. Assessing the sensitivity of regression results to unmeasured confounders in observational studies. *Biometrics* 1998; **54**(3): 948-63.
